# Supplementary material for: E-Health and M-Health in Obesity Management: A Systematic Review and Meta-Analysis of RCTs
Source: Nutrients. 2025 Jul 1;17(13):2200. doi: 10.3390/nu17132200 (PMC12251417; doi:10.3390/nu17132200)
Supplement: Supplementary file 1 [file nutrients-17-02200-s001.zip › Supplementary Materials.pdf]

Supplementary materials – Search Strategy, Table S1, Figure S1 e Figure S2

Search strategies for all databases

PUBMED:
Search: **(E-health[Title/Abstract] OR M-health[Title/Abstract]) AND (obesity)**

("E-health"[Title/Abstract] OR "M-health"[Title/Abstract]) AND ("obeses"[All Fields] OR "obesity"[MeSH Terms] OR "obesity"[All Fields] OR "obese"[All Fields] OR "obesities"[All Fields] OR "obesity s"[All Fields])

Translations

obesity: "obeses"[All Fields] OR "obesity"[MeSH Terms] OR "obesity"[All Fields] OR "obese"[All Fields] OR "obesities"[All Fields] OR "obesity's"[All Fields]

SCOPUS:

(E-health OR M-health [tiab]) AND (Obesity [tiab]) AND (trial [tiab])

Web of Science

(E-health OR M-health [topic]) AND (Obesity [topic]) AND (trial [topic])

Table S1: The risk of bias assessment, based on the five domains of the RoB 2.0 tool

	Risk of bias domains					
	D1	D2	D3	D4	D5	Overall
**Bernardo et al., 2024,**	🟢	🟢	🟡	🟢	🟢	🟡
**Choi et al, 2023**	🟢	🟢	🟢	🟢	🟢	🟢
**Domal et al, 2023**	🟢	🟢	🟡	🟢	🟢	🟡
**Riuz-Cortes et al, 2023**	🟢	🟢	🟡	🟢	🟢	🟡
**Múzquiz-Barberá et al, 2023**	🟢	🟢	🟡	🟢	🟢	🟡
**Bijlholt et al, 2021**	🟢	🟢	🟡	🟢	🟢	🟡
**Fenton et al, 2021**	🟢	🟡	🟡	🟢	🟢	🟡
**Welzel et al, 2021**	🟢	🟡	🟡	🟢	🟢	🟡
**Yu et al., 2021**	🟢	🟢	🟡	🟢	🟢	🟡
**Alencar et al., 2020**	🟢	🟢	🟢	🟢	🟢	🟢
**Duncan et al., 2020**	🟢	🟢	🟡	🟢	🟢	🟡
**Yousuful et al., 2019; HAPPY NL**	🟡	🔵	🟢	🟢	🟢	🟡
**Yousuful et al., 2019; HAPPY AZM**	🟡	🔵	🟢	🟢	🟢	🟡
**Yousuful et al., 2019; HAPPY LONDON**	🟢	🟢	🟢	🟢	🟢	🟢
**Johnson et al, 2019**	🟢	🟢	🟢	🟢	🟢	🟢
**Hutchesson et al., 2018**	🟢	🟢	🟡	🟢	🟢	🟡
**Lee et al., 2018**	🟢	🟢	🟡	🟢	🟢	🟡
**Merchart et al, 2017**	🟢	🟢	🟡	🟢	🟢	🟡
**Rader et al., 2017**	🟢	🟢	🟢	🟢	🟢	🟢
**Rogers et al.; 2016**	🟢	🟢	🟡	🟢	🟢	🟡
**Wagner et al., 2016**	🟢	🟡	🟡	🟢	🟢	🟡
**Gregosky et al., 2015**	🟡	🟡	🟢	🟢	🟢	🟡
**Tomkins Lane et al, 2015**	🔴	🟢	🟢	🟢	🟢	🔴
**Wang et al., 2015**	🟢	🟢	🟡	🟢	🟢	🟡

https://methods.cochrane.org/bias/resources/rob-2-revised-cochrane-risk-bias-tool-randomized-trials

Nejadghaderi SA, Balibegloo M, Rezaei N. The Cochrane risk of bias assessment tool 2 (RoB 2) versus the original RoB: A perspective on the pros and cons. Health Sci Rep. 2024 Jun 3;7(6):e2165. doi: 10.1002/hsr2.2165. PMID: 38835932; PMCID: PMC11147813

(D1) bias due to the *randomization process*

(D2) deviation from intended intervention

(at least 60% / differ by more than 40% or intention to treat)

(D3) missing outcome data *(20% and 10% difference between the 2 groups)*

(D4) measurement of outcomes

(D5) selection of the reported result

Overall: Judgement (High; Some Concern; Low; No information)

🔴 🟡 🟢 🔵

Figure S1: forest and funnel linked to anthropometric outcome

	ES	95% CI	N1	N2
Choi, 2024/BF%	-0.48	-1.21 , 0.26	213	241
Domal, 2023/BF%	-0.81	-1.69 , 0.07	218	246
Hutchesson, 2018/BF%	-0.72	-1.60 , 0.16	200	227
Lee, 2018/BF%	-0.79	-1.75 , 0.18	175	204
Lee, 2018/BF%1	-0.86	-1.79 , 0.07	201	225
Lee, 2018/BF%2	-0.65	-1.57 , 0.26	161	162
Rogers, 2016/BF%	-0.96	-1.56 , -0.35	214	244
Rogers, 2016/BF%1	-0.99	-2.16 , 0.19	214	243

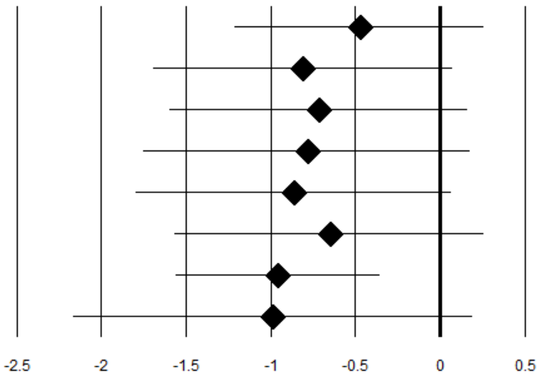

Forest BF%

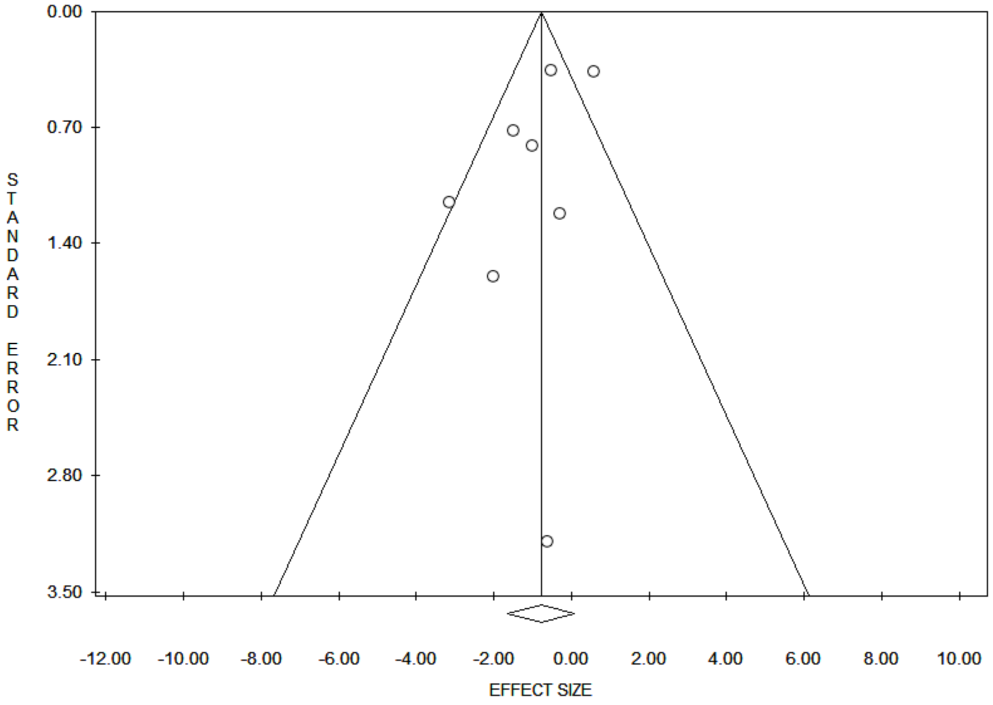

Funnel BF%

	ES	95% CI	N1	N2
Choi, 2024/BMI	-0.46	-1.21 , 0.29	483	557
Domal, 2023/BMI	-0.40	-1.17 , 0.37	488	562
Hutchesson, 2018/BMI	-0.40	-1.19 , 0.39	470	543
Johnson, 2019/BMI	-0.40	-1.14 , 0.33	488	562
Johnson, 2019/BMI1	-0.46	-1.19 , 0.28	488	562
Lee, 2018/BMI	-0.41	-1.21 , 0.38	445	520
Lee, 2018/BMI1	-0.40	-1.17 , 0.36	471	541
Lee, 2018/BMI2	-0.48	-1.24 , 0.27	431	478
Merchart, 2017/BMI	-0.08	-0.35 , 0.19	453	478
Rogers, 2016/BMI	-0.51	-1.24 , 0.22	484	560
Rogers, 2016/BMI1	-0.49	-1.25 , 0.27	484	570
Ruiz-Cortes, 2023/BMI	-0.45	-1.24 , 0.33	436	502
Wagner, 2016/BMI	-0.42	-1.18 , 0.34	428	503
Welzel, 2021/BMI	-0.42	-1.18 , 0.34	433	502
Yu, 2021/BMI	-0.45	-1.19 , 0.29	490	568

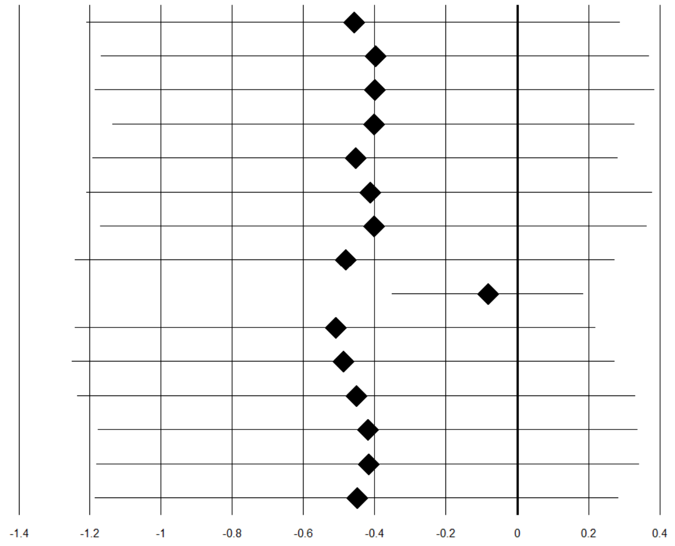

Forest BMI

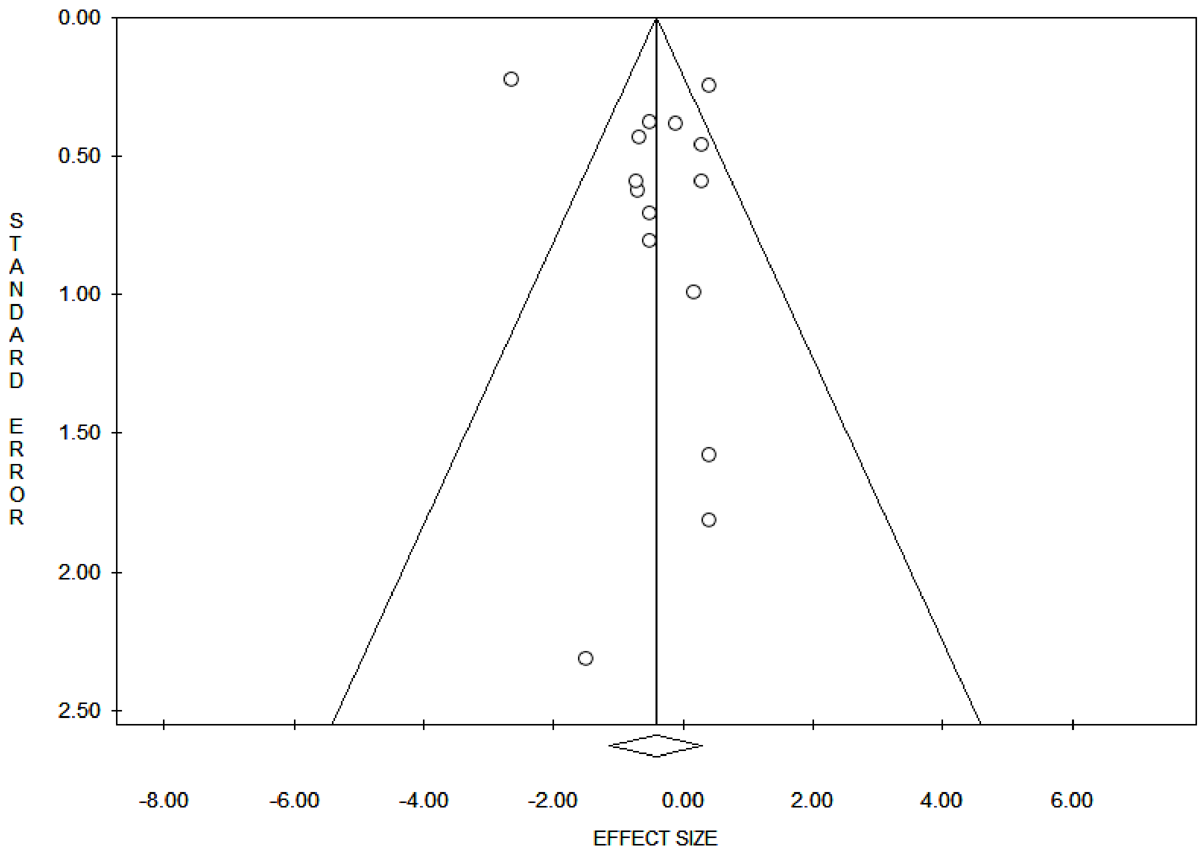

Funnel BMI

	ES	95% CI	N1	N2
Bernardo 2024/Weight	0.41	-0.46 , 1.29	398	429
Choi, 2024/Weight	0.46	-0.42 , 1.35	395	426
Domal, 2023/Weight	0.45	-0.43 , 1.33	400	431
Duncan, 2020/Weight	0.38	-0.51 , 1.26	374	402
Duncan, 2020/Weight1	0.34	-0.54 , 1.23	374	400
Hutchesson, 2018/Weight	0.25	-0.64 , 1.14	382	412
Johnson, 2019/Weight	0.40	-0.47 , 1.27	400	431
Johnson, 2019/Weight1	0.40	-0.47 , 1.28	400	431
Lee, 2018/Weight	0.65	-0.27 , 1.57	357	389
Lee, 2018/Weight1	0.50	-0.38 , 1.38	383	410
Lee, 2018/Weight2	0.58	-0.34 , 1.49	343	347
Rogers, 2016/Weight	0.07	-0.95 , 1.08	396	429
Rogers, 2016/Weight1	0.51	-0.52 , 1.53	396	428
Wagner, 2016/Weight	0.49	-0.40 , 1.37	340	372
Yu, 2021/Weight	0.42	-0.45 , 1.29	402	437

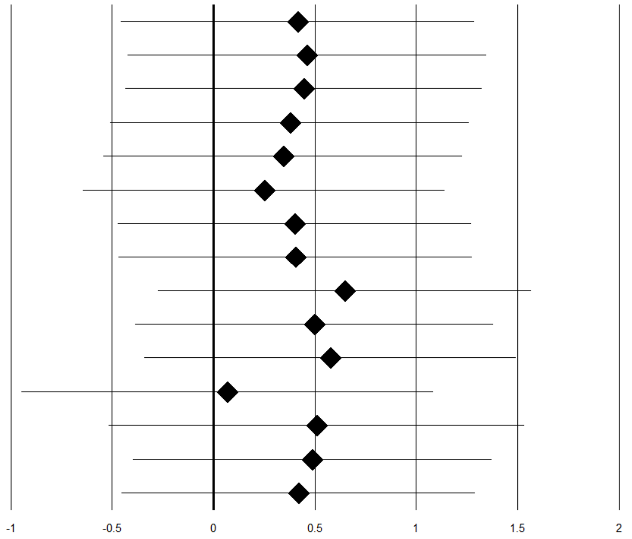

Forest Weight

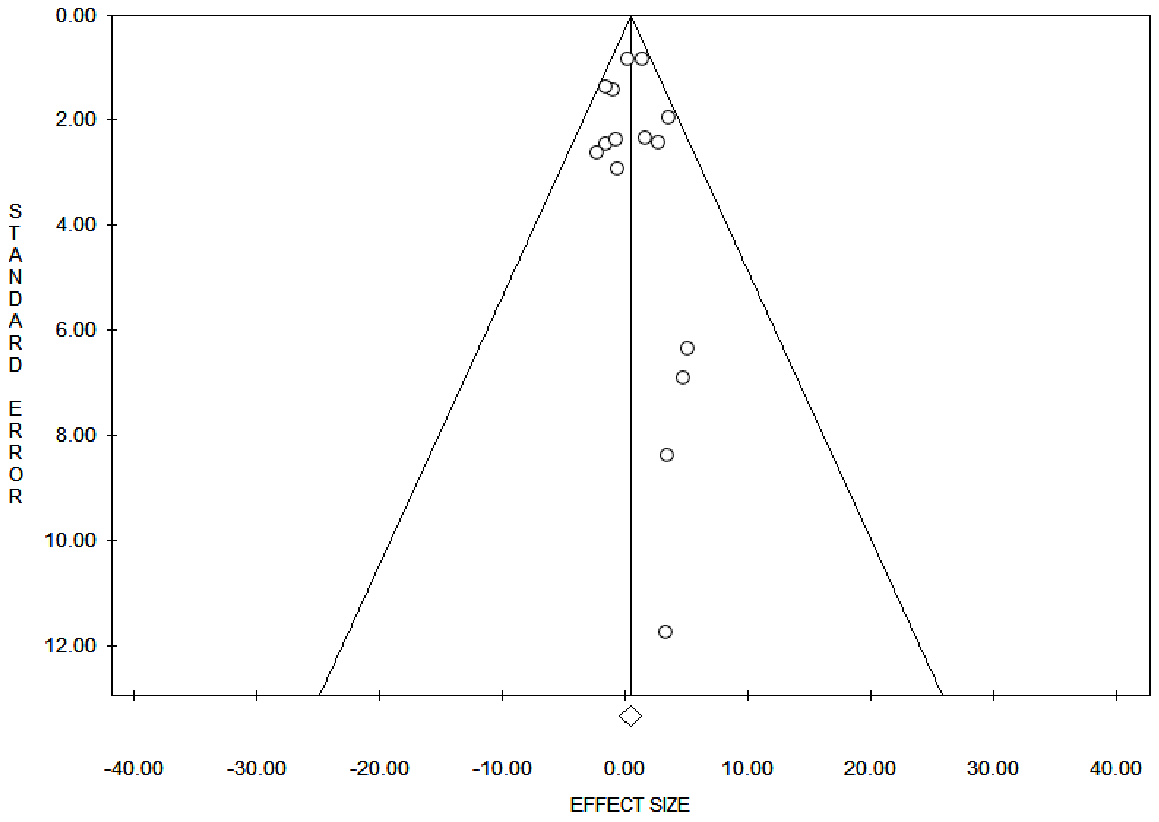

Funnel Weight

	ES	95% CI	N1	N2
Domal, 2023/Weist	-1.88	-3.31 , -0.45	239	324
Duncan, 2020/Weist	-2.02	-3.39 , -0.66	213	295
Duncan, 2020/Weist1	-2.00	-3.42 , -0.57	213	293
Lee, 2018/Weist1	-1.59	-3.03 , -0.15	222	303
Lee, 2018/Weist2	-1.85	-3.47 , -0.22	182	240
Merchart, 2017/Weist	-1.38	-2.47 , -0.28	204	240
Rogers, 2016/Weist	-2.02	-3.55 , -0.50	235	322
Rogers, 2016/Weist1	-1.50	-2.98 , -0.02	235	321

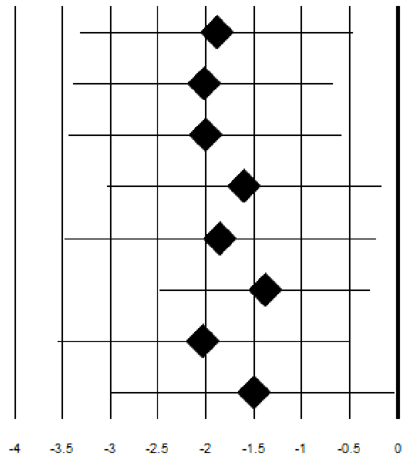

Forest Weist

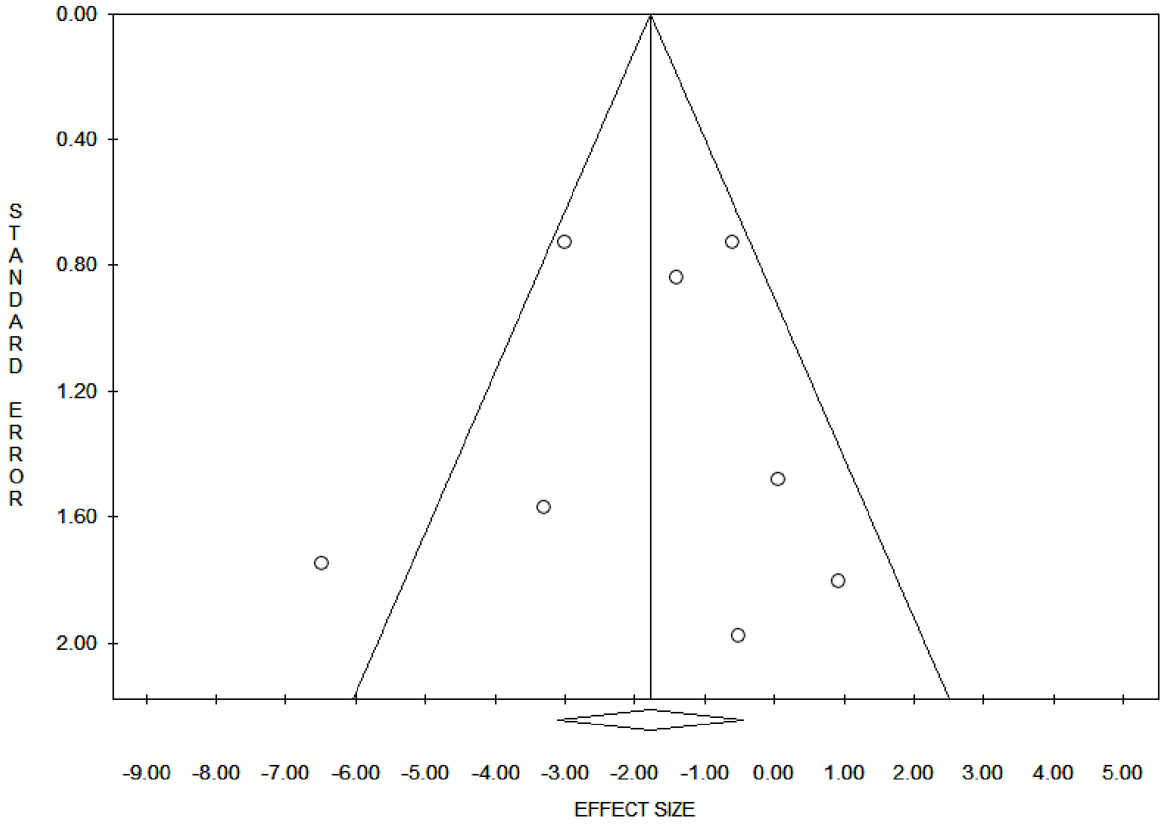

Funnel Weist

Figure S2: forest and funnel linked to lifestyle behaviours outcome

	ES	95% CI	W	N1	N2
Bijholt, 2021/Eating behavior	-0.21	-0.36 , -0.06	15.16%	337	374
Bijholt, 2021/Eating behavior1	0.00	-0.15 , 0.15	15.16%	337	374
Bijholt, 2021/Eating behavior2	0.31	0.17 , 0.46	15.16%	337	374
Rogers, 2016/Eating behavior	-0.04	-0.81 , 0.74	6.88%	14	12
Rogers, 2016/Eating behavior1	-0.05	-0.80 , 0.71	7.04%	14	13
Ruiz-Cortes, 2023/Eating behavior	0.03	-0.31 , 0.37	12.64%	62	70
Ruiz-Cortes, 2023/Eating behavior1	0.00	-0.34 , 0.34	12.64%	62	70
Ruiz-Cortes, 2023/Eating behavior2	0.04	-1.37 , 1.44	2.96%	62	2
Wagner, 2016/Eating behavior	-1.18	-1.54 , -0.82	12.36%	70	69
Overall (random-effects model)	-0.13	-0.40 , 0.14	100.00%	1295	1358

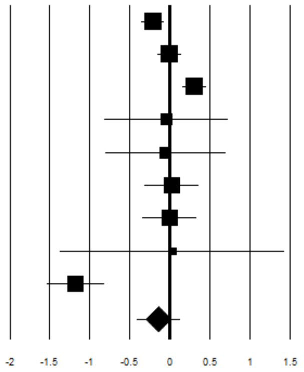

Forest eating behavior

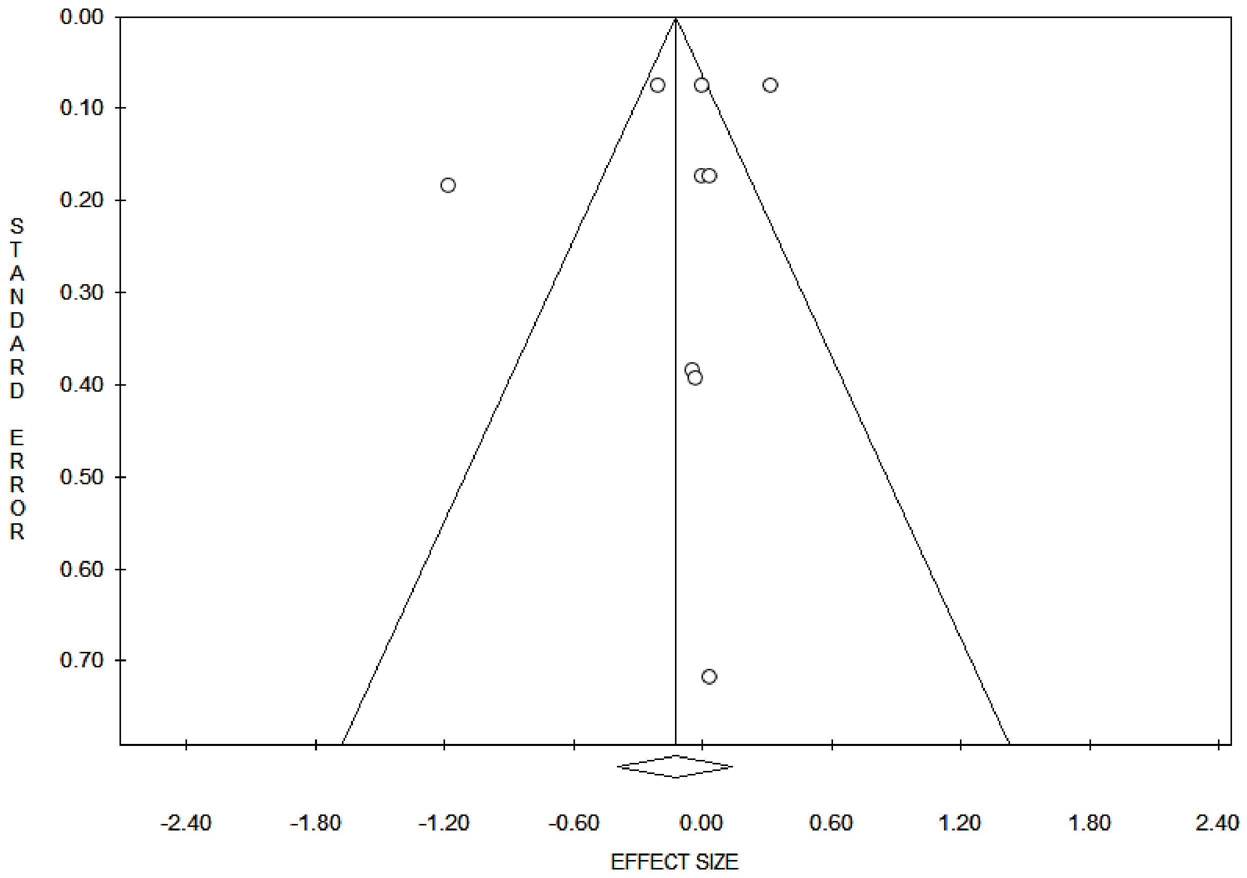

Funnel eating behavior

	ES	95% CI	W	N1	N2
Bijholt, 2021/Energy intake	-0.22	-0.37 , -0.08	22.21%	378	377
Fenton, 2021/Energy intake	-4.23	-5.22 , -3.25	17.96%	17	37
Hutchesson, 2018/Energy intake	-0.22	-0.74 , 0.30	20.90%	28	29
Rogers, 2016/Energy intake	-0.12	-0.89 , 0.65	19.42%	14	12
Rogers, 2016/Energy intake1	-0.29	-1.05 , 0.47	19.51%	14	13
Overall (random-effects model)	-0.93	-1.88 , 0.01	100.00%	451	468

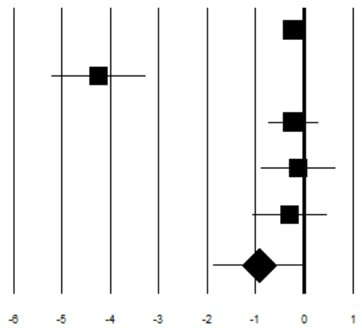

Forest energy intake

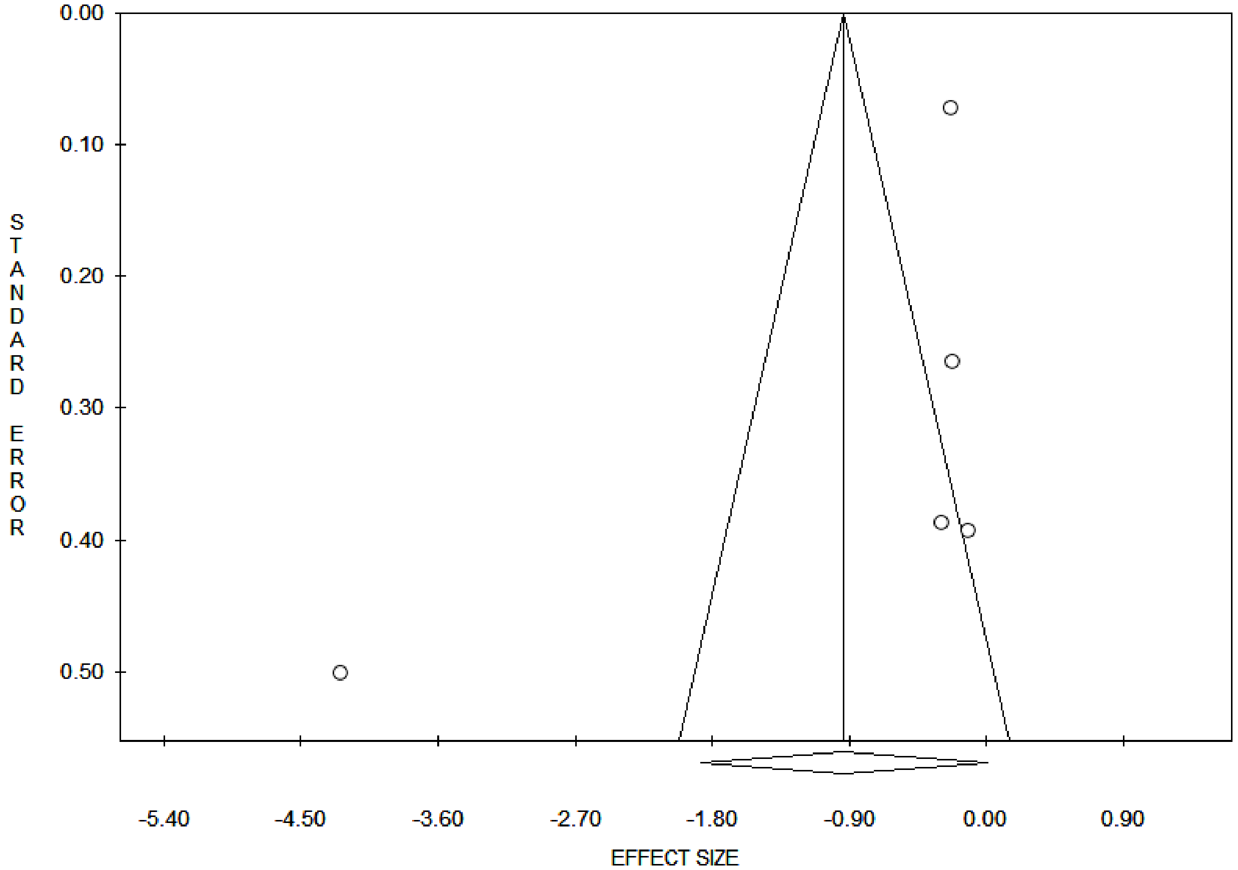

Funnel Energy Intake

	ES	95% CI	W	N1	N2
Bijholt, 2021/Physical activity	0.11	-0.04 , 0.26	12.55%	344	340
Fenton, 2021/Physical activity	-0.77	-1.24 , -0.29	10.60%	36	37
Hutchesson, 2018/Physical activity	-0.25	-0.77 , 0.27	10.24%	28	29
Johnson, 2019/Physical activity	1.15	0.21 , 2.10	7.01%	10	10
Muzquiz-Barbera, 2023/Physical activity	0.10	-0.25 , 0.44	11.56%	62	70
Rogers, 2016/Physical activity	-0.68	-1.48 , 0.11	8.11%	14	12
Rogers, 2016/Physical activity1	0.77	-0.01 , 1.55	8.19%	14	13
Ruiz-Cortes, 2023/Physical activity	0.05	-0.30 , 0.39	11.57%	62	70
Wang, 2015/Physical activity	1.12	0.59 , 1.65	10.16%	33	30
Wang, 2015/Physical activity1	-1.40	-1.95 , -0.85	10.00%	33	30
Overall (random-effects model)	-0.01	-0.39 , 0.36	100.00%	636	641

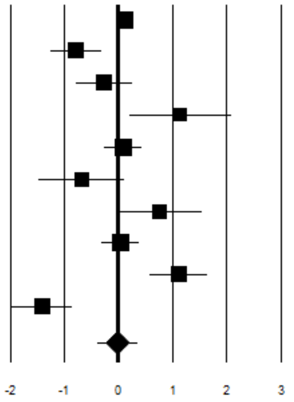

Forest Physical Activity

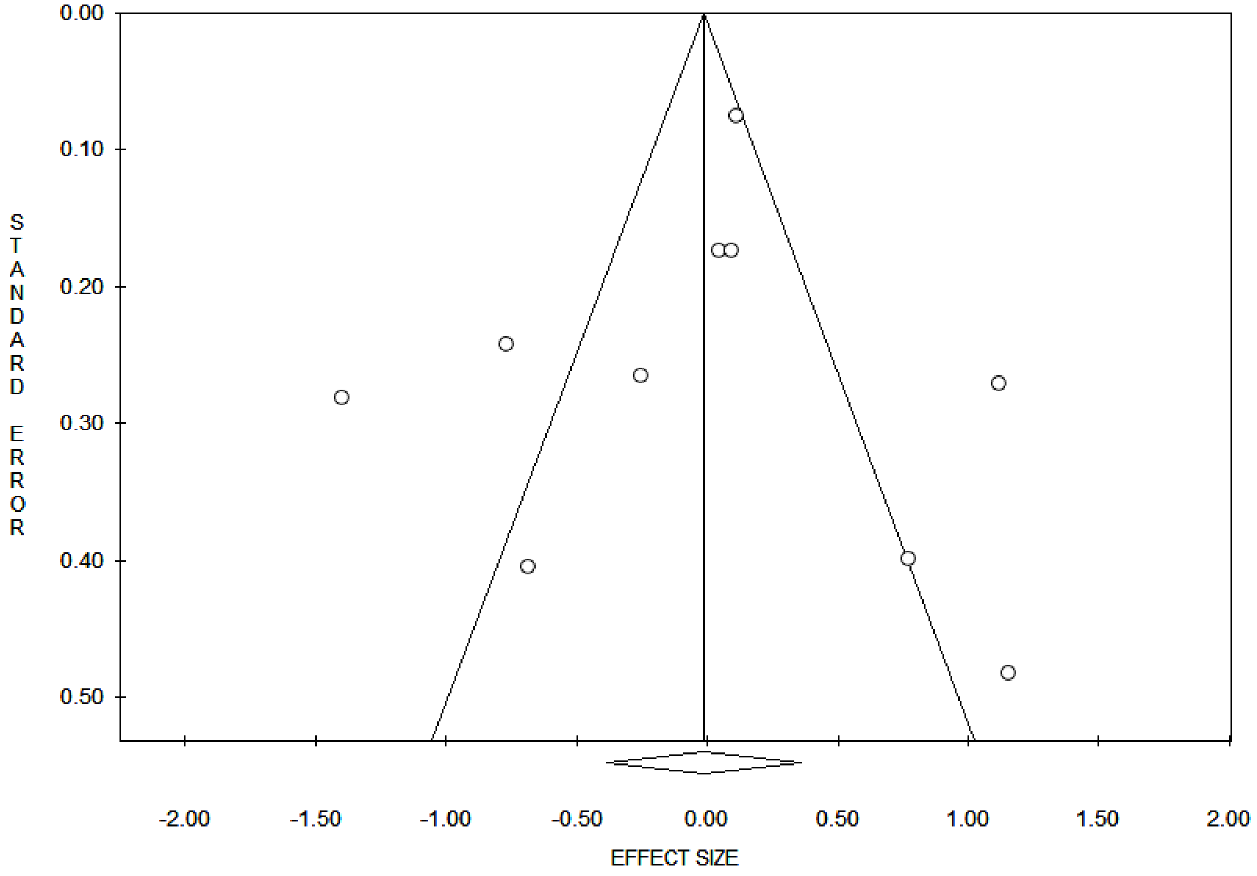

Funnel Physical Activity
